# Quantitative polymerase chain reaction in paucibacillary leprosy diagnosis: A follow-up study

**DOI:** 10.1371/journal.pntd.0007147

**Published:** 2019-03-05

**Authors:** Raquel R. Barbieri, Fernanda S. N. Manta, Suelen J. M. Moreira, Anna M. Sales, José A. C. Nery, Lilian P. R. Nascimento, Mariana A. Hacker, Antônio G. Pacheco, Alice M. Machado, Euzenir M. Sarno, Milton O. Moraes

**Affiliations:** 1 Leprosy Laboratory, Oswaldo Cruz Institute—Fiocruz, Rio de Janeiro, Rio de Janeiro, Brazil; 2 PROCC—Programa de Computação Científica—Fiocruz, Rio de Janeiro, Rio de Janeiro, Brazil; Institut Pasteur, FRANCE

## Abstract

**Objective:**

The diagnosis of paucibacillary (PB) leprosy cases remains a challenge because of the absence of a confirmatory laboratory method. While quantitative polymerase chain reaction (qPCR) has been shown to provide reliable sensitivity and specificity in PB diagnoses, a thorough investigation of its efficacy in clinical practice has not yet been published. The present study evaluated patients with suspected leprosy skin lesions by using qPCR to identify PB individuals in the Leprosy Outpatient clinic at the Oswaldo Cruz Foundation in Rio de Janeiro, Brazil.

**Methods:**

One hundred seventy-two suspected PB cases were included in the study. The patients were evaluated by a dermatologist at three different times. The clinical dermato-neurological examination and collected samples were performed on the first visit. On the second visit, the results of the histopathological analysis and PCR assay (DNA-based *Mycobacterium leprae* qPCR-targeting 16S gene) results were analyzed, and a decision regarding multi-drug therapy was made. A year later, the patients were re-examined, and the consensus diagnosis was established.

**Results:**

In 58% (100/172) of cases, a conclusive diagnosis via histopathological analysis was not possible; however, 30% (30/100) of these cases had a positive PCR. One hundred ten patients (110/172) attended the third visit. The analysis showed that while the sensitivity of the histopathological test was very low (35%), a qPCR alone was more effective for identifying leprosy, with 57% sensitivity.

**Conclusion:**

The use of qPCR in suspected PB cases with an inconclusive histology improved the sensitivity of leprosy diagnoses.

## Author summary

Leprosy is a major public health problem in several low- to middle-developed countries where a consistent number of new cases are diagnosed every year. Irrespective of the use of efficient treatment, diagnosis is late because of the absence of a gold standard method. Thus, the multi-drug therapy (MDT) is unable to control of disease transmission even though early diagnosis, adequate MDT treatment of new cases, and contact surveillance have been considered the pillars of leprosy endemic control. Quantitative polymerase chain reaction (qPCR)-based *Mycobacterium leprae* DNA direct detection in clinical samples has been developed to support diagnosis. This study used qPCR to differentially diagnose suspected cases of paucibacillary leprosy and to follow them after treatment to confirm the consensus diagnosis and therapeutic approaches. It was concluded that qPCR and histopathological analysis increase clinical diagnosis accuracy, and qPCR has higher sensitivity. The routine use of qPCR laboratory tests could therefore improve the precision of early diagnosis for the achievement of leprosy control.

## Introduction

Prerequisites for leprosy control, early detection, and adequate treatment are crucial for reducing the risk of disability and interrupting transmission, thereby decreasing the burden of the disease. Leprosy diagnoses are based on clinical manifestations, the presence of acid-fast bacilli (AFB) determined through microscopy in slit skin smears, and histopathological analyses of skin biopsy samples. Nevertheless, diagnosis is challenging, particularly in the early stages and in a paucibacillary (PB) form characterized by the absence of bacilli in slit skin smears. In addition, skin macules or plaques, frequently found in PB leprosy, represent the clinical manifestations of many other dermatoses, such as pityriasis alba, rosacea granulomatosa, granuloma anulare, cutaneous tuberculosis, and sarcoidosis.

Histological diagnosis of leprosy is indicated by two cardinal features: neural aggression and bacillar identification [1]. In PB, however, histological changes can simply be a perivascular lymphohistiocytic infiltrate or an epithelioid granuloma, a nonspecific alteration in the dermis that hinders a differential histopathological diagnosis [2,3]. In a paper published almost 30 years ago, Lucas and Ridley stated that “the difficulty and lack of agreement in diagnosing early PB leprosy by histopathology is frustrating.” They asserted that “there is no reason why the essentially subjective nature of histological observation and diagnosis should change in the future”[4].

Indeed, no clinical or histopathology tests are capable of either confirming or rejecting all suspected PB cases. In the last two decades, studies have demonstrated the potential of polymerase chain reaction (PCR) technology for the rapid detection and identification of leprosy in clinical specimens [5–8]. More recently, real-time PCR has improved the rate of disease detection by facilitating the direct quantitation of the bacterial DNA content in clinical samples, thereby increasing the reliability of the results [9–11]. This tool has become particularly important in the differential diagnosis of cases with a negative bacillary load and non-conclusive histopathology [12–14].

In the absence of a gold standard, previous studies have used clinical diagnoses or histopathologic analyses as reference standards for evaluating PCR sensitivity and specificity. However, the inherent subjectivity of these methods underscores the need for more reliable criteria for defining reference standards.

Since 2012, qPCR has been used as a support in the clinical and histopathological leprosy diagnostic procedures at our clinic. The present study aimed to provide more precise sensitivity and specificity for qPCR in PB leprosy. Suspected PB cases were followed for a year, and consensus diagnoses were established after observation and evaluation of the presence or absence of skin lesions one year later. The consensus diagnosis became the reference standard for analyzing the role of qPCR in PB leprosy diagnoses in clinical practice.

## Methods

This prospective observational study, nested in a clinic cohort, was performed at the Leprosy Outpatient Clinic of the Oswaldo Cruz Foundation (Fiocruz), a national reference center for leprosy in Rio de Janeiro, RJ, Brazil. All individuals referred to the clinic for diagnosis, from January 2012 through December 2016, presenting with suspicious PB leprosy skin lesions were included.

Excluded were all cases exhibiting a positive bacteriological index (BI) on a slit skin smear, visualized bacilli in the first histopathological analysis, and suspicion of relapse. Also excluded were those cases suspected of having the pure neural form without skin lesions.

**Fig 1:**
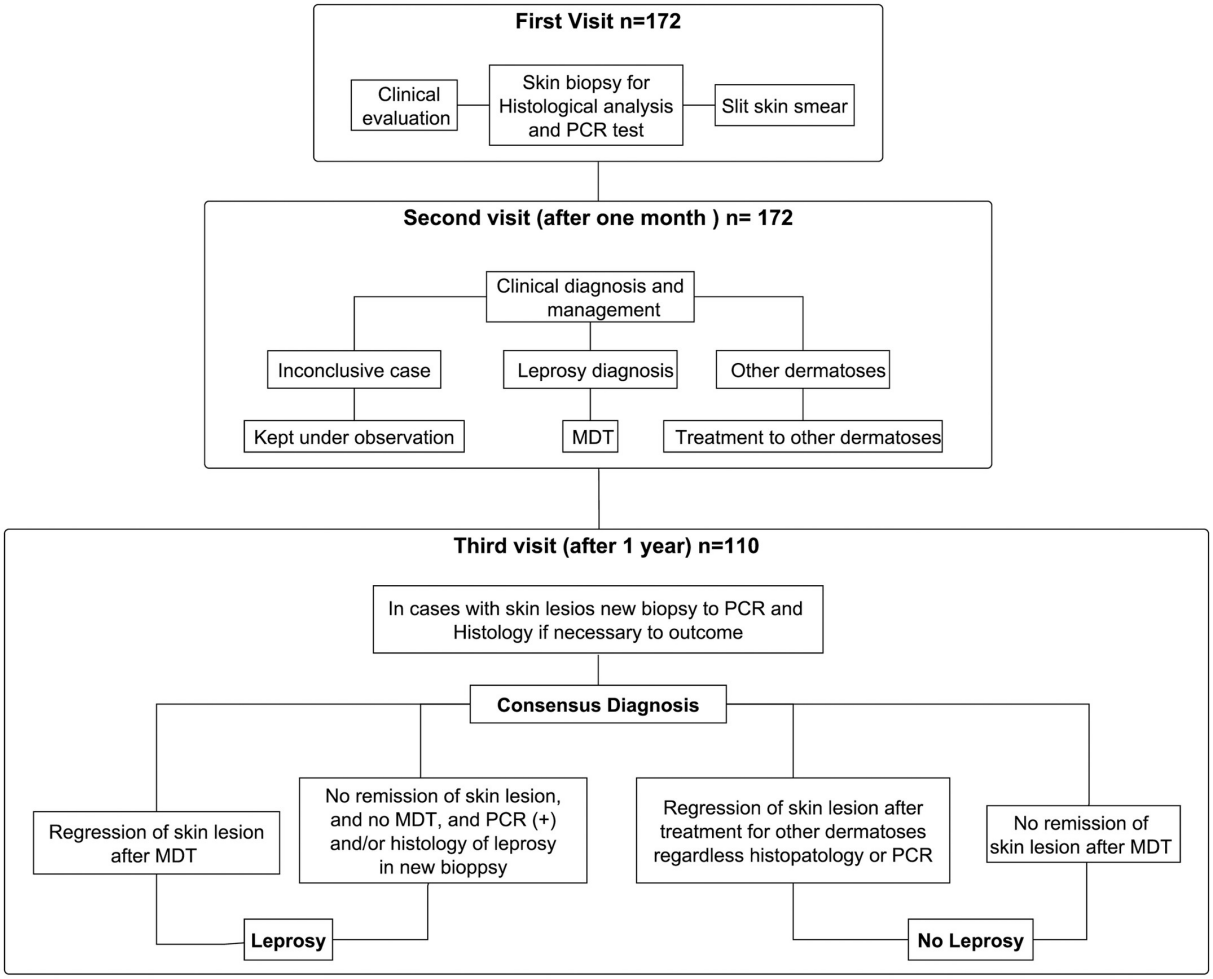
Clinical evaluations of the cases. A clinical evaluation and sample collection at the first visit; the histopathological and PCR results and the decision on pursuing MDT in the second; the consensus diagnosis in the third visit one year after the second.

Each patient had three evaluations. The first included a clinical evaluation and sample collection (skin biopsy for histology and qPCR). In the second evaluation one month later, the histopathological and PCR results were analyzed, and a decision regarding MDT was made. At the third visit one year later, the consensus diagnosis was established (Fig 1). For each patient who continued to experience skin lesions, a new biopsy was performed for histological examination and PCR if necessary. These three-step evaluations are detailed below.

### First visit: Initial clinical dermato-neurological examination and sample collection

#### Clinical-epidemiological data and bacillary analysis

All patient clinical-epidemiological data, including age, gender, contact with leprosy patients, and lesion duration, were collected and recorded on the standard outpatient form. The symptoms and the clinical description of the skin lesions, i.e., number, color, size, location, and type (macula or plaque), including lesion sensitivity, were recorded after the dermatological and physiotherapeutic examinations. Slit skin smears were taken from four sites (the ear lobes, one elbow, and a cutaneous lesion) for bacteriological diagnosis. One skin biopsy was performed (punch 6 mm) from suspected lesions for a histopathological analysis and a PCR assay. These skin samples were divided into two parts. One was stored in formalin for histological study, and the other, in 70% ethanol for *Mycobacterium* (*M*.) *leprae* DNA detection.

#### Histopathological analysis

One skin biopsy sample was prepared for routine histopathological examination, hematoxilin-eosin, and Wade staining. All the cases were analyzed by an experienced dermatopathologist and were classified as either PB leprosy, other dermatoses, or nonspecific histological features (NSHFs). The cases were categorized as PB leprosy in the presence of an inflammatory lymphohistiocytic or granulomatous infiltrate in the nervous fillets and/or the erector muscle of the hair [1]. If the histopathological alterations typical of other skin diseases were detected, the cases were classified as “other dermatoses.” Those in which the histopathological changes were nonspecific were categorized as “NSHFs.”

#### Polymerase chain reaction assays

For DNA extraction, the DNeasy blood and tissue kit (Qiagen, Valencia, CA, USA) was used in accordance with the specifications. The qPCR technique is standardized to amplify the 16S rRNA *M*. *leprae*-specific target through the use of the TaqMan amplification assay. All the reactions were performed in triplicate through the same real-time PCR system (Applied Biosystems StepOne). The results were obtained in accordance with the first fluorescent signal detection, cycle threshold (Ct) [10]. As recommended by previous studies and in accordance with the laboratory’s standard operating procedures for validating clinical molecular tests, the sample was considered positive when it exhibited Ct </ = 38.5 (3 genomes) in at least two of the three reactions (triplicates) [10,15,16].

### Second visit: Laboratorial results and clinical management

At the second visit, the dermatologist used the Ministry of Health clinical criteria for leprosy diagnosis (regardless of the histopathological and PCR results) [17]to categorize all the cases and to define the treatment as follows: (i) Leprosy patients began treatment with PB MDT, (ii) cases diagnosed with other dermatosis were referred to a general dermatological service for treatment, and (iii) inconclusive cases were kept under observation. All of the patients were scheduled for a third clinical evaluation one year after the second visit.

### Third visit: Follow-up

All of the patients were invited for a third clinical examination during which the consensus diagnosis was established. The presence or absence of skin lesion and the results of the PCR and histological analysis performed during the first visit were considered. If the lesions remained unchanged, a new biopsy was performed for diagnostic clarification through PCR and histopathological analysis. The cases were classified as either leprosy or non-leprosy (consensus diagnosis) according to the following criteria:

**Leprosy cases**
- Patients with skin lesion remission on the third visit post-MDT; and
- Patients who on the third visit had skin lesions without MDT and those whose new biopsy results showed the histopathological characteristics of leprosy or a positive PCR.

**Non-leprosy cases**
- Patients with skin lesions on the third visit despite receiving MDT;
- Patients with lesion remission on the third visit after specific treatment for other dermatoses; and
- Patients with lesion remission on the third visit without MDT or any other medication.

After the third visit, the consensus diagnosis (leprosy or non-leprosy) was established as the reference standard.

### Statistical analysis

The frequencies for the clinical-epidemiological characteristics were ascertained through the use of proportions for the categorical variables and centrality and dispersion measures for the continuous variables. The proportions of the histological diagnostic (leprosy, other dermatosis, or NSHFs) and the PCR results (positive or negative) were calculated and compared by use of the chi-square test (significance level of 5%). The frequency distribution of the cases on the basis of the related qPCR, histopathological tests, and consensus diagnoses was described through a flow chart. The sensitivity and specificity of the histology, the qPCR, and the combination of these two diagnostic methods were also calculated. The confidence intervals were calculated at a level of 95%.

### Ethics approval

This study was approved by the Ethics in Research Committee of the Oswaldo Cruz Foundation CAAE: 38053314.2.0000.5248, number: 976.330–10/03/2015. Written informed consent was received from all the participants and the parents or guardians of the children under 18 years old included in the study.

## Results

As is shown in Table 1, the lesions in 65% of the cases were recent, having evolved within a year or less. Most patients had only one lesion (Table 1). The mean age was 42.2 (+/− 17.8 SD), and the range was 15–79.

**Table 1:** Clinical-epidemiological characteristics: N = 172.

Variable	Frequency	%
*Gender*		
Female	108	63.0
Male	64	37.0
*Household contact*		
Yes	80	46.6
No	92	53.4
*Skin lesion*		
Macula	110	64.0
Plaque	62	36.0
*Progression time*		
≤12 months	112	65.0
13–24 months	41	24.0
>24 months	19	11.0
*Number of lesions*		
1 lesion	107	62.0
2 lesions	27	16.0
≥3 lesions	38	22.0
*MDT treatment initiation*		
Yes	76	44.0
No	96	56.0

The results of the histopathological and PCR tests in the skin samples indicated that it was impossible to reach a leprosy diagnosis via histology in 58% (100/172) of all cases. In addition, among those with NSHFS, 30% had a positive PCR (Table 2).

**Table 2:** Proportions for histological diagnosis and polymerase chain reaction results.

		Histological diagnosis			
		Leprosy	Other dermatoses	NSHFs*	Total
**PCR**	**Positive**	12 (48%)	8 (17%)	30 (30%)	50
	**Negative**	13 (52%)	39 (83%)	70 (70%)	122
	Total	25	47	100	172

*Nonspecific histological features

**Fig 2:**
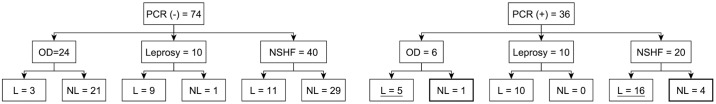
Flow chart of quantitative polymerase chain reaction results, histopathological results, and consensus diagnosis. The 21 underlined patients had exhibited the histological features of other dermatoses or NSHFs, and the consensus diagnosis was leprosy (L). These cases were diagnosed by PCR. The five highlighted cases were false PCR positives. L = consensus diagnosis of leprosy; NL = consensus diagnosis of non-leprosy.

The frequency distribution for the qPCR results, histopathology diagnosis, and consensus diagnosis is presented for the 110 cases that made the third visit (110/172). The consensus diagnosis of almost half of this group (54/110) was leprosy (Fig 2).

Only one of the five cases with a false positive PCR (highlighted in Fig 2) presented with plaque skin lesions and a granulomatous infiltrate histologically diagnosed as granulomatous rosacea. This patient was treated with doxycycline 200 mg/day for seven months after which remission of the cutaneous lesion was observed. The remaining four cases presented with macular lesions and NSHFs in the second evaluation. All were household contacts. Despite the patients’ positive PCR results and epidemiological histories, clinical progress was monitored because of the patients’ atypical maculae. Spontaneous healing of the cutaneous lesions was observed in these four cases.

**Fig 3:**
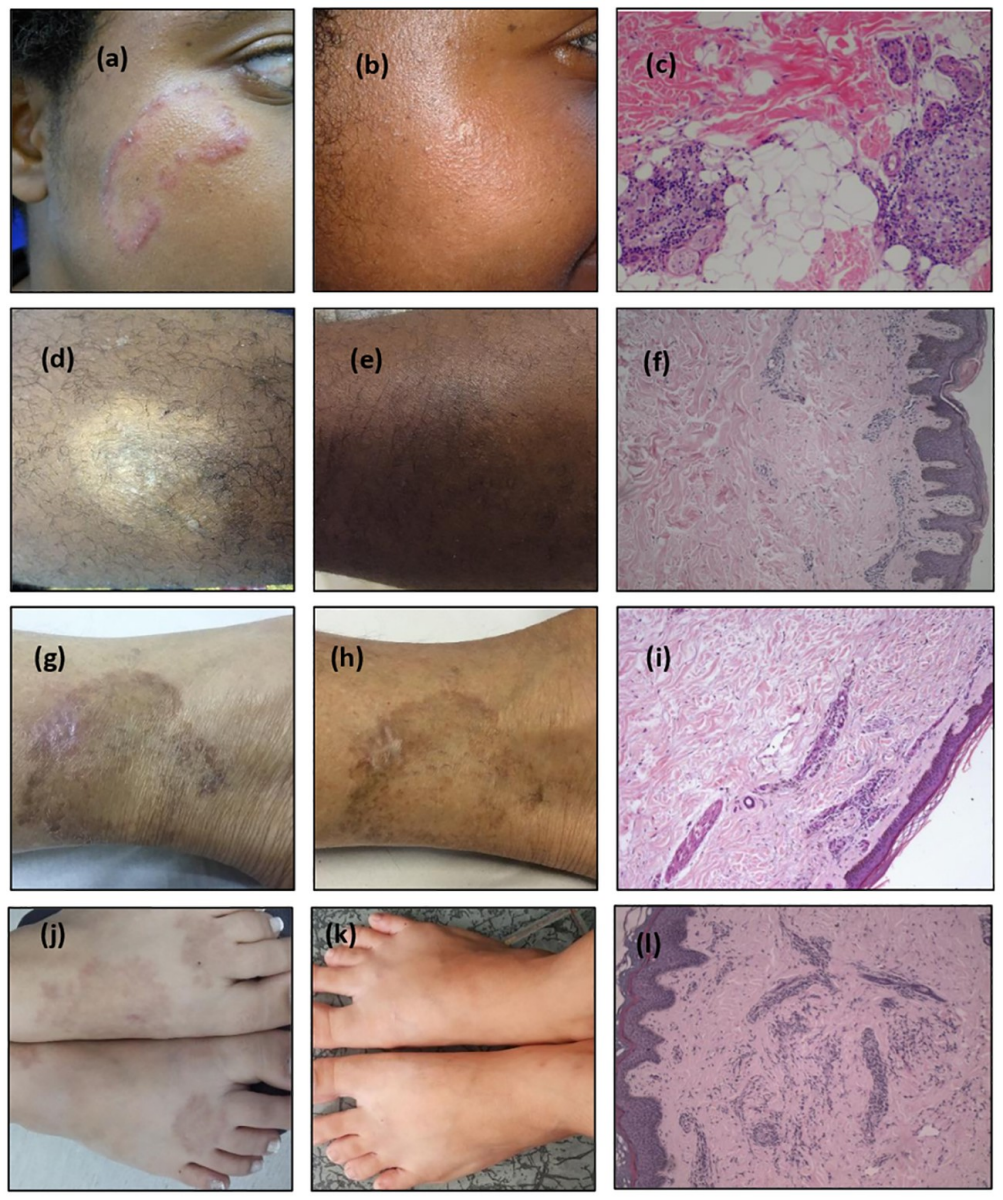
Examples of cases diagnosed as other dermatoses via histology and for which polymerase chain reaction was decisive in the leprosy diagnosis. All the cases were qPCR positive. **Case I**: (A) before multi-drug therapy (MDT), (B) one year after MDT; (C) histology: hematoxilin-eosin (H.E.) 10X, granulomatous rosacea: nerve bundles with normal appearance and granulomas attached to adnexa; **Case II**: (D) before MDT, (E) 8 months after MDT, (F) histology: H.E. 20X, suggestive of eczema; **Case III**: (G) before MDT, (H) residual lesion **at release** from treatment, (I) histology: H.E. 10X, **nonspecific** infiltrated inflammatory; **Case IV**: (J) before MDT, (K) one year after the end of MDT, (L) histology: HE 10X, granuloma annulare.

During the second visit, 21 patients (underlined in Fig 2) whose consensus diagnosis was leprosy were observed to have been diagnosed with other dermatoses via histology; thus, the qPCR results were decisive in establishing the diagnosis. Fig 3 illustrates four of these 21 leprosy cases identified by qPCR. It shows the histopathological features and skin lesions before and after MDT.

To analyze histological sensibility and specificity, the two non-leprosy groups were analyzed together, i.e., the NSHFs were added to the cases with other dermatoses. The data demonstrated that despite the high specificity, histopathological sensitivity was very low in this sample. Indeed, qPCR alone was more effective in identifying leprosy because PCR sensitivity (57%) was 22% higher than histopathology sensitivity (35%) (Table 3).

**Table 3:** Histological and quantitative polymerase chain reaction (qPCR) sensitivity, specificity, and accuracy, and the combination of these two diagnostic methods.

Histological results	Leprosy	Non-Leprosy	Total	Sensitivity (IC 95%)	Specificity (IC 95%)	Accuracy (IC 95%)
Leprosy	19	1	20	35% (22.2–47.9)	98% (94.8–1.01)	67% (58.8–76.2)
NSHFs**+ Other dermatoses	35	55	90			
Total	54	56	110			
**qPCR results**						
Positive	31	5	36	57% (44.2–70.5)	91% (81.5–97.4)	75% (65.7–82.0)
Negative	23	51	74			
Total	54	56	110			
**Histology + PCR**						
Histo* leprosy + NSHFs** with qPCR (+)	35	5	40	65% (52.0–77.5)	91% (81.2–97.6)	78% (66.3–88.6)
Histo OD** + NSHFs** with qPCR (−)	19	51	70			
Total	54	56	110			

Histo* = histology; OD** = other dermatoses; NSHFs** = nonspecific histological features

## Discussion

The results of this study made clear that for some patients whose clinical and histological changes were insufficient for reaching a diagnosis, qPCR aided in establishing leprosy. In the cases in which the PB leprosy was suspected, the simultaneous use of qPCR and histology increased the sensitivity of a PB diagnosis without a loss of specificity.

A majority of the cases evaluated in this study had one skin lesion, and most of these were the macula type. Moreover, most patients reported having noticed their lesions one year or less before their initial visit. Nayar et al. affirmed the near impossibility of visualizing inflammation in the nervous fillets by histopathology during the first year of the disease [18]. The results of the present study demonstrated that whenever typical leprosy features or changes in other dermatoses were present, the sensitivity and specificity of the histopathological analyses were high. However, the analysis of the NSHFs cases, half of the sample, revealed very low sensitivity.

Studies have found qPCR sensitivity ranges of 34%–80% in PB patients [9–11]. However, compared to the sensitivity for histopathology, even this relatively low sensitivity can be considered a step forward for the differentiation of early leprosy from other dermatoses. In this sample, which was composed of suspected PB cases under investigation in our clinic, qPCR sensitivity was greater than histopathological sensitivity. This demonstrates that in clinical practice, *M*. *leprae* detection by qPCR in patients with a negative bacillary load and NSHFs would be of great value for limiting subjectivity in the diagnosis of PB leprosy.

One of the five apparently false positive PCR cases had tuberculoid granulomas. Because the histopathological diagnosis was granulomatous rosacea, the patient was treated with doxycycline at a general dermatological clinic. Surprisingly, the rosacea plaque cleared only after seven months of daily use of the antibiotic. Doxycycline and minocycline are antibiotics in the tetracycline group with the same mechanism of action. For decades, minocycline, which has a strong bactericidal action against *M*. *leprae* [19], was used as an alternative leprosy treatment and It was found to be efficacious [20–21]. A possible explanation for the delay in lesion remission is that this patient, who was still being monitored, actually had PB leprosy and not granulomatous rosacea.

Three explanations are possible for the four positive PCR cases in which the lesions cleared without MDT or any other medication. It should be noted that studies using qPCR-targeting 16S and other genes have described a 100% specificity for PB leprosy [9,10]. In addition, some PB cases have been found to progress to spontaneous healing [1,22]. It can therefore be hypothesized that the leprosy lesions of these patients might have been cured spontaneously. Another explanation is that these patients might have had a positive PCR because of a subclinical infection because all four were household contacts. The third possibility is that these were false-positive cases and that their skin lesions were cutaneous manifestations of other dermatoses. Nevertheless, none of the above-mentioned studies performed follow-up post-diagnoses. The 100% specificities in the PB cases were obtained through the reference standard of results of a histopathological examination and/or clinical diagnosis at a particular point in time.

Leprosy is a slow-progressing disease for which a gold standard for diagnosis does not exist. Therefore, defining the consensus diagnosis after observing the progression of the cases, as was done in the present study, was an additional step forward in providing higher precision for the PCR sensitivity and specificity measures. The sample size could be considered a limitation even though it appears that no other study has yet analyzed more than 100 PB patients for molecular diagnosis [23].

The present study clearly demonstrated that even for experienced dermatologists and pathologists, the difficulties encountered in interpreting the clinical and histopathological differential diagnosis of PB cases may be responsible for many delayed diagnoses. Because of the integration of leprosy control actions into the delivery of general health services in Brazil and other endemic countries, patients will continue to escape detection because few clinicians have the skills, experience, or expertise to diagnose the disease, especially in the early stages [24].

In addition, studies have highlighted that any declaration of the elimination of leprosy as a public health problem results in the neglect of ongoing disease control strategies, such as household surveillance, community awareness campaigns, and training for health professionals. Consequently, the lower the incidence, the less prepared will be clinicians and pathologists to identify leprosy in their clinical practice [25,26]. Brazil offers an example. In some states in which the disease had been under control, a recent trend of the diagnosis of new cases with disabilities, a late diagnostic indicator, has been observed [27,28].

In the last decade, real-time PCR has proved to be particularly useful in three areas: the identification of the drug-resistant bacilli used for treating leprosy [29], differential diagnoses for distinguishing between reaction and relapse by viability analyses of the bacillus [30], and the facilitation of the use of conventional diagnostic methods in difficult cases [23,31]. Although this technology is becoming accessible, faster, and cheaper, the infrastructure, such as equipment and trained professionals, is still a barrier to implementing qPCR in resource-limited settings. Portable real-time machines need to be tested and validated. In this regard, the GeneXpert has been used for pulmonary tuberculosis diagnosis in primary care facilities where unspecialized health care professionals are conducting the assays with the same sensitivities [32]. Sample processing technologies are available for *M*. *leprae* DNA detection; however, reference centers are still necessary.

In conclusion, the use of qPCR in suspected PB cases with a non-conclusive histology improved the sensitivity of leprosy diagnoses. Because early diagnosis is crucial to avoiding transmission and incapacitating neural lesions and achieving the goal of a leprosy-free world, the need for tools for the detection and confirmation of the disease among suspected individuals wherever they may live is indeed urgent.

## Supporting information

S1 Checklist STROBE checklist. (DOC)
